# TELEHEALTH UTILIZATION BY RURAL OLDER CANCER SURVIVORS: A COMMUNITY-BASED QUALITATIVE ASSESSMENT

**DOI:** 10.1093/geroni/igad104.0751

**Published:** 2023-12-21

**Authors:** Marquita Lewis-Thames, Shawn Trimble, Francesca Rosen

**Affiliations:** Northwestern Feinberg School of Medicine, Chicago, Illinois, United States; Northwestern University Feinberg School of Medicine, Chicago, Illinois, United States; Northwestern University Feinberg School of Medicine, Chicago, Illinois, United States

## Abstract

In a nationwide survey, rural clinic uptake of telehealth utilization increased by 70% compared to pre-pandemic utilization. Despite advancements in tele-delivered cancer care for rural residents, older adults are slower to engage the technology; yet examinations of the challenges rural older cancer survivors face when incorporating telehealth into complex cancer care are limited. Guided by the Andersen Healthcare Utilization Model, we qualitatively examined barriers and facilitators of telehealth utilization for rural older cancer survivors. Ongoing interviews began in March 2022. We purposively sampled telehealth-using rural older cancer survivors, caregivers of rural older cancer survivors, and healthcare professionals that served this population. The interview guide probed participants regarding telehealth utilization for cancer and survivorship care. We used thematic analysis and deductively coded to the Andersen Model constructs. Preliminary findings are based on six rural older cancer survivors, caregivers, and healthcare professionals. Most participants were Non-Hispanic White, had at least some college, traveled greater than 31 minutes to an oncologist, and used their cell phone or computer to access telehealth. We coded similar perceptions between survivors, caregivers, and healthcare providers. Emergent themes included (1) Telehealth provided critical and necessary services, (2) Existing technological literacy and infrastructure enabled telehealth uptake, (3) Geographic isolation related to the rural environment impaired participant’s ability to access telehealth-delivered care, and (4) Real-time provider-patient communication at critical points in participants’ care facilitated telehealth utilization. Overall, participants reported that telehealth was useful for overcoming barriers common to rurality and often a suitable strategy to deliver cancer care.

